# Epidemiology and Pathogen Shift of Tinea Capitis: A Comparative Analysis of Adults and Children in Nanchang, China (2022–2024)

**DOI:** 10.1111/myc.70142

**Published:** 2025-12-22

**Authors:** Qian Peng, Afang Xu, Qijing Xiao, Zhihua Li, Qing Jiang, Yangmin Gao, Yun Jin, Yunpeng Luo, Xinyi Fan, Rui Xu, Jiao Xu, Wenjin Ai, Xiaobing Wang

**Affiliations:** ^1^ Dermatology Hospital of Jiangxi Province, Jiangxi Provincial Clinical Research Center for Skin Diseases, Candidate Branch of National Clinical Research Center for Skin Diseases, JXHC Key Laboratory of Skin Infection and Immunity The Affiliated Dermatology Hospital of Nanchang University Nanchang China; ^2^ Affiliated Dermatological Hospital, Jiangxi Medical College Nanchang University Nanchang China

**Keywords:** asymptomatic infections, China, dermatophytes, epidemiology, *Microsporum canis*, risk factors, tinea capitis, *Trichophyton violaceum*

## Abstract

**Background:**

Tinea capitis remains a public health concern, especially in children, with evolving trends. Data from Nanchang, China, regarding comparative analyses between adults and children is limited.

**Objectives:**

This study aimed to analyze the clinical and epidemiological characteristics, pathogen spectrum, risk factors, and household transmission of tinea capitis in Nanchang from 2022 to 2024, comparing features between adult and pediatric patients.

**Methods:**

A single‐center retrospective study was conducted involving 239 patients (170 children, 69 adults) diagnosed with confirmed tinea capitis. Demographic, clinical, and risk factor data were collected. Pathogens were identified via microscopy, culture, and molecular methods.

**Results:**

Significant increases were observed in adult cases (28.87% vs. historical 9.73%), particularly among postmenopausal women. Anthropophilic pathogens dominated overall (65.42%), but zoophilic species increased significantly (32.71% vs. historical 16.96%). Clinical types differed: black dot tinea predominated in adults (81.16%), while kerion (42.94%) was more common in children. Animal contact was a key risk factor for children (55.29%). Household transmission occurred in 22.90% of surveyed families, with 100% pathogen concordance and frequent asymptomatic adult carriers (50% of affected households).

**Conclusions:**

Notable differences exist between children and adult tinea capitis in Nanchang, with trends showing increasing adult cases and a shift towards zoophilic pathogens. Prevention strategies should be tailored to specific age groups and transmission modes, emphasising household screening and management of human and animal sources.

## 1 Introduction

Tinea capitis is a common superficial fungal infection of the scalp and hair caused by dermatophytes. It has a global distribution and exhibits a higher incidence in children [1, 2]. Children's increased susceptibility is attributed to their immature immunity, underdeveloped skin barrier, and low sebum antifungal activity. In contrast, adults are more resistant due to robust immunity and a higher sebum antifungal content [3]. Transmission occurs primarily through direct contact with infected humans or animals, or indirectly via contaminated fomites, which can lead to outbreaks in households and schools [2, 4].

Clinical manifestations vary depending on the causative species, host susceptibility, and the degree of inflammatory response. The clinical spectrum ranges from asymptomatic carriage or mild pruritus and scaling to severe inflammatory reactions like kerion. Notably, kerion may lead to permanent scarring alopecia and impose significant psychological burden [5]. Common clinical classifications include grey patch, black dot, kerion, and favus [6].

Despite the widespread use of griseofulvin and public health improvements that have substantially reduced the incidence, tinea capitis remains a persistent public health challenge in many developing regions [7]. In China, the distribution of tinea capitis has distinct regional characteristics [8]. Previous studies report that zoophilic *Microsporum canis* predominates in eastern, western, and northeastern China, while anthropophilic *Trichophyton violaceum* is most common in central regions.

Nanchang, the capital of Jiangxi Province in central China, has a humid subtropical monsoon climate conducive that favours fungal growth. Historical data from the region indicate that *Trichophyton schoenleinii* was the primary pathogen of tinea capitis sixty years ago. Due to sustained control efforts, *T. schoenleinii* has nearly been eradicated, and anthropophilic *T. violaceum* has emerged as the dominant pathogen [9]. However, recent comprehensive studies on the epidemiology of tinea capitis in Nanchang—particularly those comparing adults and children and examining household transmission—are scarce. Therefore, this study aimed to analyse the clinical and epidemiological characteristics, pathogen spectrum, risk factors, household transmission, and comparative analysis between adult and paediatric patients with tinea capitis in Nanchang from 2022 to 2024, so as to provide evidence for targeted prevention and control strategies.

## 2 Methods

### 2.1 Study Design and Participants

This single‐center retrospective descriptive study was conducted at the Dermatology Hospital of Jiangxi Province. We enrolled patients diagnosed with tinea capitis between January 2022 and December 2024. The diagnostic confirmation required direct microscopic examination of hyphae or spores, or the isolation of dermatophytes through fungal culture. Patients with other scalp disorders, such as psoriasis, alopecia areata, seborrheic dermatitis, neurodermatitis, or trichotillomania were excluded. Participants were categorised into two groups: children (< 18 years) and adults (≥ 18 years).

### 2.2 Data Collection

Demographic and clinical data were gathered using a standardised questionnaire. Collected variables included age, sex, residence, disease duration, clinical type, history of animal contact, history of exposure to persons with dermatophytosis, concomitant superficial fungal infections, underlying diseases, laboratory results (direct microscopy and fungal culture), in addition to clinical and mycological data from household members. The study was approved by the hospital's ethics committee (Approval No. KY2025‐35‐01), and written informed consent was obtained from all participants or their guardians.

### 2.3 Mycological Examination

Specimens of hair, scales, or pus were collected from lesions. One portion underwent examination by fluorescence microscopy; the detection of hyphae or spores was considered a positive result. Another portion was cultured on Sabouraud Glucose Agar (SGA) slants at 28°C for up to 14 days. Species were preliminarily identified based on their macroscopic and microscopic morphological characteristics. For morphologically ambiguous strains, molecular identification was performed by PCR amplification of the ITS region using primers ITS1 (5′‐TCCGTAGGTGAACCTGCGG‐3′) and ITS4 (5′‐TCCTCCGCTTATTGATATGC‐3′). The amplified products were sequenced and analysed by BLAST against the NCBI database. A strain was identified as the same species as a reference if both sequence identity and query coverage exceeded 99%. Given the high morphological similarity between *Nannizzia gypsea* and *Nannizzia incurvata*, all isolates presumptively identified as *Nannizzia* genus were also subjected to this molecular confirmation. The obtained ITS sequences were deposited in GenBank with the accession numbers provided below.

### 2.4 Statistical Analysis

Data were analysed using IBM SPSS Statistics v27.0. Categorical variables were compared using the chi‐square test or Fisher's exact test when sample sizes were small (*n* < 40) or if more than 20% of the cells had an expected count below 5. Post hoc analysis was performed using adjusted standardised residuals. A *p*‐value below 0.05 was considered statistically significant.

## 3 Results

### 3.1 Demographic and Clinical Characteristics

A total of 239 patients with confirmed tinea capitis were included for analysis. The annual case distribution was as follows: 2022 (21.3%, 51/239), 2023 (39.7%, 95/239), and 2024 (38.9%, 93/239) suggesting a considerable rise in incidence after the initial year, potentially linked to the easing of COVID‐19‐related public health measures.

**FIGURE 1:**
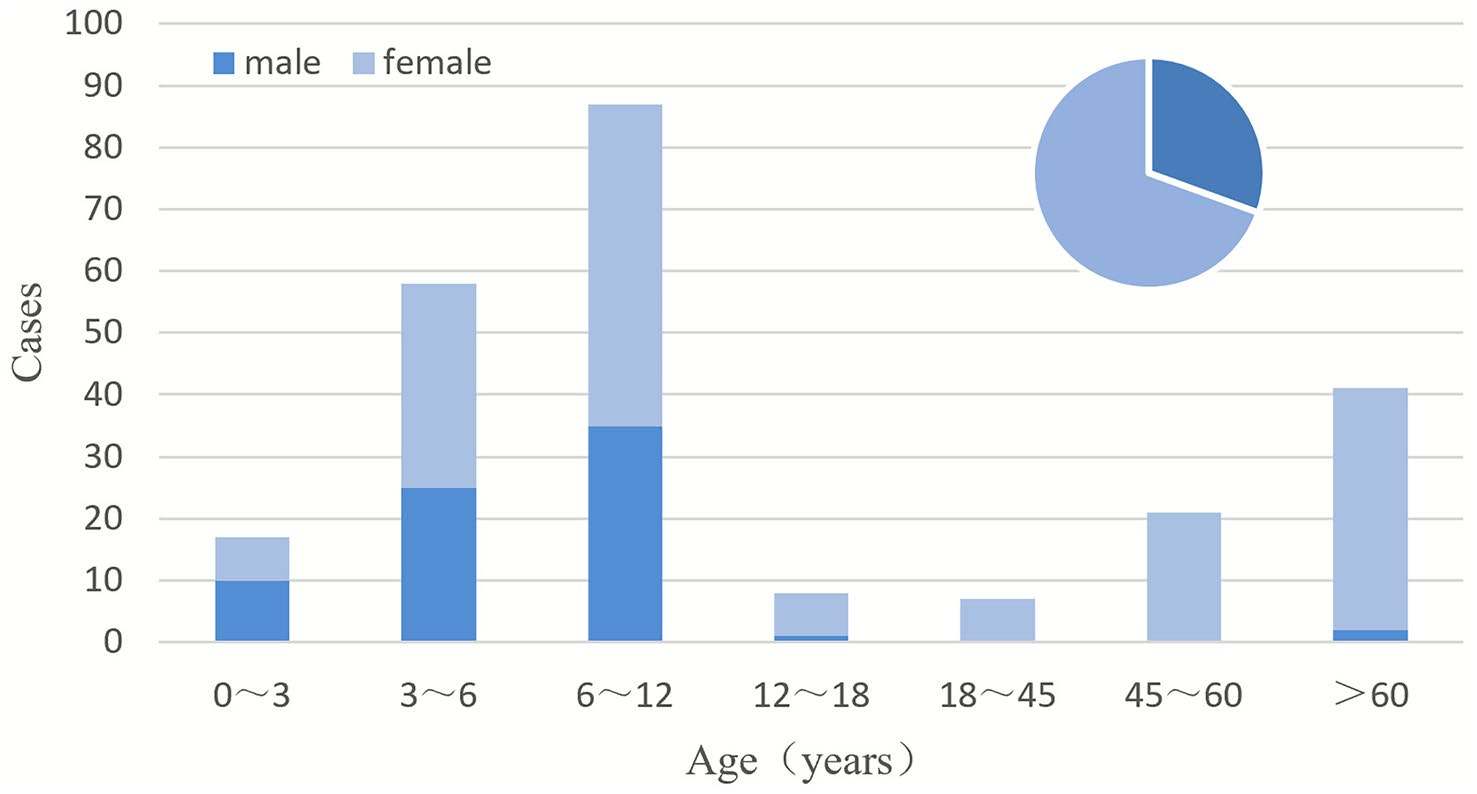
Age and gender distribution of patients.

Patient ages ranged from 4 months to 84 years. Children constituted 71.13% (170/239) of the cohort, with a median age of 6 years; adults accounted for 28.87% (69/239), with a median age of 63 years. The overall mean age was 21.89 ± 26.06 years (The age distribution of patients is shown in Table 1). The incidence rate was highest in the 6–12‐year age group, declined thereafter, but rose again after age 45, mainly among elderly women (Figure 1). The overall female‐to‐male ratio was 2.27:1 (166:73). This skew towards females was markedly more evident in adults (F:M = 67:2, ratio 33.5:1) than in children (F:M = 99:71, ratio 1.39:1) (*p* < 0.001; Table 2). The number of urban patients was significantly higher than that of rural patients (74.48% vs. 25.52%). Furthermore, the proportion of rural patients was significantly higher among adults than children (*p* < 0.001).

**TABLE 1:** Age distribution of patients (*n* = 239).

Age group (years)	Number	Percentage
≤ 3	17	7.11
3–6	58	24.27
6–12	87	36.40
12–18	8	3.35
18–45	7	2.93
45–60	21	8.79
> 60	41	17.15

**TABLE 2:** Demographic and clinical characteristics by age group.

Characteristic	Total (*n* = 239)	Children (*n* = 170)	Adults (*n* = 69)	*p*
Gender, *n* (%)				
Male	73 (30.54)	71 (41.76)	2 (2.90)	< 0.001
Female	166 (69.46)	99 (58.24)	67 (97.10)	
Residence, *n* (%)				
Urban	178 (74.48)	140 (82.35)	38 (55.07)	< 0.001
Rural	61 (25.52)	30 (17.65)	31 (44.93)	
Disease duration (months), *n* (%)				
≤ 1	75 (31.78)	65 (38.46)	10 (14.93)	< 0.001
1–3	45 (19.07)	36 (21.30)	9 (13.43)	0.165
3–12	61 (25.85)	45 (26.63)	16 (23.88)	0.664
> 12	55 (23.31)	23 (13.61)	32 (47.76)	< 0.001

**FIGURE 2:**
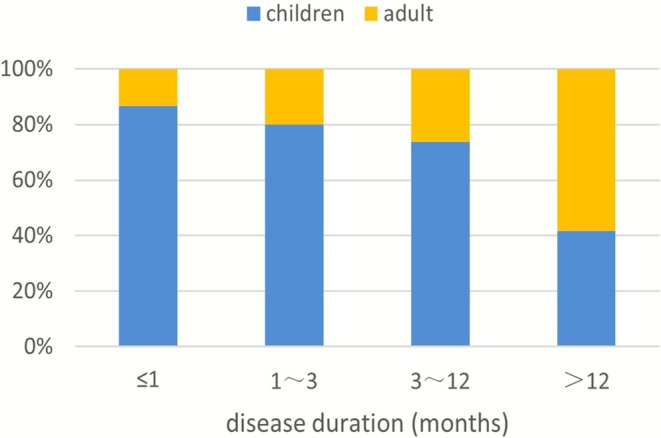
Disease duration distribution by age group.

Data on disease duration (from symptom onset to first medical consultation) were available for 236 patients (169 children, 67 adults). The duration ranged from 2 days to 50 years, with a mean of 23.73 ± 70.74 months and a median of 3 months. The most frequent duration category was ≤ 1 month (31.78%, 75/236) (Table 2). Disease duration was significantly associated with age: adults had a significantly higher proportion of cases with a duration > 12 months compared to children (*p* < 0.001), whereas children had a significantly higher proportion of cases with a duration of ≤ 1 month (*p* < 0.001). The distribution of disease duration by age groups is shown in Figure 2.

### 3.2 Mycological Findings and Pathogen Distribution

All patients underwent direct microscopic examination and fungal culture. The positivity rate for direct microscopy was 99.6% (238/239). The sole microscopy‐negative case was a 6‐year‐old boy; however, *M. canis* was isolated from his culture, possibly due to prior treatment with oral terbinafine. The fungal culture positivity rate was 90.38% (216/239). Among the culture‐positive samples, 213 isolates (98.61%, 213/216) were identified based on macroscopic and microscopic morphology. The remaining three isolates (1.39%, 3/216), which displayed ambiguous morphological features, were identified by DNA sequencing of the ITS region as *T. violaceum* , *T. mentagrophytes*, and *T. tonsurans* (accession numbers: PX499633, PX499634, PX499635, respectively). Similarly, the four isolates (1.85%, 4/216) preliminarily identified as *Nannizzia* genus were confirmed by ITS sequencing to be *Nannizzia gypsea* (accession numbers: PX499636, PX499637, PX499638, PX499639, respectively). In total, six species were isolated. Two cases (0.93%, 2/216) presented with mixed infections and were consequently excluded from the subsequent analysis of pathogen distribution. Thus, 214 cases with single infection (150 children, 64 adults) were included in the analysis.

**FIGURE 3:**
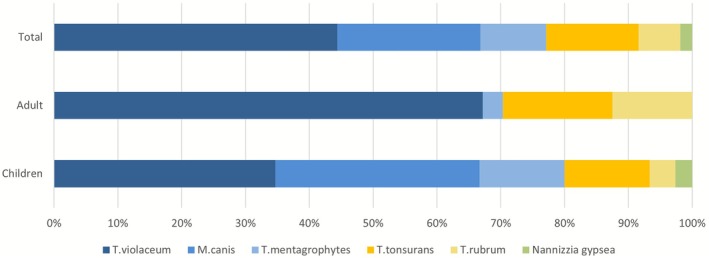
Pathogen distribution by age group.

Anthropophilic dermatophytes ( *T. violaceum* , *T. tonsurans*, *T. rubrum* ) accounted for 65.42% (140/214) of infections, while zoophilic dermatophytes ( *M. canis* and *T. mentagrophytes*) accounted for 32.71% (70/214). The overall pathogen distribution is shown in Figure 3. *T. violaceum* was the predominant species (44.39%, 95/214), followed by *M. canis* (22.43%, 48/214), *T. tonsurans* (14.49%, 31/214), *T. mentagrophytes* (10.28%, 22/214), *T. rubrum* (6.54%, 14/214), and *Nannizzia gypsea* (1.87%, 4/214).

Although anthropophilic dermatophytes represented the primary cause of infection in both children and adults, the specific pathogen profile differed markedly between the two age groups (Table 3). The prevalence of zoophilic dermatophyte infections was significantly higher in children than in adults (*p* < 0.001). Conversely, anthropophilic infections predominated in adults, accounting for a significantly greater proportion compared to children (*p* < 0.001). At the species level, infections caused by *T. violaceum* (*p* < 0.001) and *T. rubrum* (*p* < 0.05) were significantly more frequent in adults. In contrast, *M. canis* (*p* < 0.001) and *T. mentagrophytes* (*p* < 0.05) were more commonly observed in children than in adults.

**TABLE 3:** Pathogen distribution and clinical types by age group.

Variable	Total (*n* = 214)	Children (*n* = 150)	Adults (*n* = 64)	*p*
Pathogen group, *n* (%)				
Anthropophilic	140 (65.42)	78 (52.00)	62 (96.88)	< 0.001
Zoophilic	70 (32.71)	68 (45.33)	2 (3.13)	< 0.001
Geophilic	4 (1.87)	4 (2.67)	0 (0.00)	0.320
Pathogen species, *n* (%)				
*T. violaceum*	95 (44.39)	52 (34.67)	43 (67.19)	< 0.001
*T. tonsurans*	31 (14.49)	20 (13.33)	11 (17.19)	0.463
*T. rubrum*	14 (6.54)	6 (4.00)	8 (12.50)	0.032
*M. canis*	48 (22.43)	48 (32.00)	0 (0.00)	< 0.001
*T. mentagrophytes*	22 (10.28)	20 (13.33)	2 (3.13)	0.024
*N. gypsea*	4 (1.87)	4 (2.67)	0 (0.00)	0.320
Clinical type, *n* (%)				
Grey patch	40 (16.74)	40 (23.53)	0 (0.00)	< 0.001
Black dot	113 (47.28)	57 (33.53)	56 (81.16)	< 0.001
Kerion	86 (35.98)	73 (42.94)	13 (18.84)	< 0.001

### 3.3 Correlation Between Clinical Type and Pathogen

Black dot was the most prevalent clinical type (47.28%, 113/239), followed by kerion (35.98%, 86/239) and grey patch (16.74%, 40/239). The distribution of clinical types differed significantly between children and adults (Table 3). Black dot was most frequently observed in adults (81.16%, 56/69), with a significantly higher prevalence than in children (*p* < 0.001). In contrast, kerion was the predominant type in children (42.94%, 73/170), and both kerion and grey patch showed significantly higher rates in children than in adults (*p* < 0.001 for both). Fisher's exact test revealed a significant association between pathogen species and clinical type (*p* < 0.001), with a strong correlation strength (Cramer's *V* = 0.643) (Table 4). Post hoc analyses using adjusted standardised residuals demonstrated that grey patch was primarily associated with *M. canis* (*p* < 0.001); black dot with *T. violaceum* (*p* < 0.001) and *T. tonsurans* (*p* < 0.01), and kerion with *T. mentagrophytes* (*p* < 0.001) and *T. rubrum* (*p* < 0.05). For infections caused by the same pathogen species, the resulting clinical type did not differ significantly between children and adults (*p* > 0.05) (Table S1).

**TABLE 4:** Clinical types of tinea capitis caused by different pathogens (*n* = 214).

Pathogen	Grey patch	Black dot	Kerion
*T. violaceum*	2 (−5.5)***	75 (7.9)***	18 (−4.0)***
*T. tonsurans*	2 (−1.8)	22 (2.7)**	7 (−1.4)
*T. rubrum*	0 (−1.8)	6 (−0.4)	8 (2.0)*
*M. canis*	33 (10.3)***	0 (−7.6)***	15 (−0.3)
*T. mentagrophytes*	1 (−1.8)	1 (−4.4)***	20 (6.1)***
*N. gypsea*	1 (0.4)	0 (−2.0)*	3 (1.8)
Total	39	104	71

*Note:* Adjusted standardised residuals in parentheses.

* *p* < 0.05.

** *p* < 0.01.

*** *p* < 0.001.

### 3.4 Temporal Shifts in Epidemiology and Pathogen Profile

Compared to historical data from 2006 to 2014 [9], this study revealed significant increases in the proportions of adult cases (*p* < 0.001), female patients (*p* = 0.003), and patients from urban areas (*p* < 0.001). The proportions of cases presenting as grey patch and kerion increased significantly (*p* < 0.001), while the proportion of black dot decreased significantly (*p* < 0.001). A significant decline was observed in the proportion of infections caused by anthropophilic dermatophytes (*p* = 0.013), whereas a substantial increase was observed for zoophilic dermatophytes (*p* < 0.001). At the species level, the proportions of *T. tonsurans* and *M. canis* rose significantly (*p* < 0.001 for both), while a notable decrease was observed for *T. violaceum* (*p* < 0.001). No significant temporal changes were observed in the proportions of *T. rubrum* , *T. mentagrophytes*, or *N. gypsea* (Table 5).

**TABLE 5:** Epidemiological and mycological shifts: 2006–2014 vs. 2022–2024.

Variable	2006–2014 (*n* = 627)	2022–2024 (*n* = 239)	*p*
Age group, *n* (%)			
Children	566 (90.27)	170 (71.13)	< 0.001
Adults	61 (9.73)	69 (28.87)	
Gender, *n* (%)			
Male	261 (41.69)	73 (30.54)	0.003
Female	365 (58.31)	166 (69.46)	
Residence, *n* (%)			
Urban	105 (16.75)	178 (74.48)	< 0.001
Rural	522 (83.25)	61 (25.52)	
Clinical type, *n* (%)			
Grey patch	20 (3.19)	40 (16.74)	< 0.001
Black dot	477 (76.08)	113 (47.28)	< 0.001
Kerion	124 (19.78)	86 (35.98)	< 0.001
Favus	6 (0.96)	0 (0.00)	0.195
Pathogen group, *n* (%)			
Anthropophilic	466 (81.47)	140 (65.42)	0.013
Zoophilic	97 (16.96)	70 (32.71)	< 0.001
Geophilic	9 (1.57)	4 (1.87)	0.900
Pathogen species, *n* (%)			
*T. violaceum*	394 (68.88)	95 (44.39)	< 0.001
*T. tonsurans*	41 (7.17)	31 (14.49)	< 0.001
*T. rubrum*	25 (4.37)	14 (6.54)	0.124
*M. canis*	15 (2.62)	48 (22.43)	< 0.001
*T. mentagrophytes*	82 (14.34)	22 (10.28)	0.286
*Nannizzia gypsea*	8 (1.40)	4 (1.87)	0.513
Other	7 (1.22)	0 (0.00)	0.201

### 3.5 Comparison of Risk Factors Between Children and Adults

The distribution of potential risk factors is summarised in Table 6. Concomitant superficial fungal infections were observed in 22.18% (53/239) of patients. Tinea corporis was the most common concurrent type (60.38%, 32/53). The prevalence of concomitant superficial fungal infections did not differ significantly between children and adult patients (*p* = 0.106). A history of contact with individuals with dermatophytosis was reported by 8.37% (20/239) of patients, with no significant difference between the two age groups (*p* = 0.907). Animal contact was identified as an important risk factor (49.37%, 118/239), which was significantly more common in children (55.29%, 94/170) than in adults (34.78%, 24/69) (*p* = 0.004). However, the rate of animal exposure did not differ significantly between male (54.79%, 40/73) and female (46.99%, 78/166) patients (*p* = 0.266).

**TABLE 6:** Risk factors by age group.

Risk factor, *n* (%)	Total (*n* = 239)	Children (*n* = 170)	Adults (*n* = 69)	*p*
Concomitant superficial fungal infections	53 (22.18)	33 (19.41)	20 (28.99)	0.106
Patient contact	20 (8.37)	14 (8.24)	6 (8.70)	0.907
Animal contact	118 (49.37)	94 (55.29)	24 (34.78)	0.004
Underlying disease	23 (9.62)	15 (8.82)	8 (11.59)	0.510

The causative pathogens showed significant associations with specific types of animal exposure (*p* < 0.001), with a moderate strength of correlation (Cramer's *V* = 0.495) (Table 7). Contact with cats was significantly associated with infections caused by *M. canis* (*p* < 0.001), while exposure to rabbits was significantly associated with *T. mentagrophytes* infections (*p* < 0.001). However, a history of animal exposure was not invariably associated with the isolation of zoophilic dermatophytes. Underlying diseases were present in 16.32% (39/239) of patients, with no significant difference between children (15.29%, 26/170) and adults (18.84%, 13/69) (*p* = 0.510).

**TABLE 7:** Association between animal contact and pathogen (*n* = 78).

Animal	*T. violaceum*	*T. tonsurans*	*T. rubrum*	*M. canis*	*T. mentagrophytes*
Cat	2 (−3.5)***	1 (−0.4)	0 (−1.3)	24 (6.5)***	0 (−3.0)**
Dog	12 (1.9)	2 (0.8)	2 (1.3)	5 (−2.4)*	4 (−0.3)
Rabbit	3 (−1.0)	0 (−1.0)	0 (−0.8)	1 (−2.8)**	10 (5.8)***
Hamster	1 (0.5)	0 (−0.3)	0 (−0.3)	1 (0.3)	0 (−0.7)
Chicken	7 (3.0)**	1 (0.9)	1 (1.2)	0 (−2.6)**	0 (−1.5)
Cow	1 (1.4)	0 (−0.2)	0 (−0.2)	0 (−0.8)	0 (−0.5)

*Note:* Adjusted standardised residuals in parentheses.

* *p* < 0.05.

** *p* < 0.01.

*** *p* < 0.001.

### 3.6 Household Transmission

Household surveys were conducted for 131 index cases (114 children, 17 adults). Tinea capitis was detected in at least one additional family member in 22.90% (30/131) of the households investigated. The secondary attack rate within households did not differ significantly between children (24.56%, 28/114) and adults (11.76%, 2/17) (*p* = 0.389). The most commonly affected family members were mothers, grandmothers, and siblings. Asymptomatic carriers were identified in 50% (15/30) of affected households, all of whom were adults. Among symptomatic cases, black dot was the predominant clinical type (93.33%, 14/15), followed by kerion (6.67%, 1/15). Twenty‐nine strains were isolated from family members. *T. violaceum* was the most prevalent (58.62%, 17/29), followed by *T. tonsurans* and *M. canis* (20.7% each, 6/29). Perfect pathogen concordance (100%, 29/29) was observed between index cases and their infected household contacts.

## 4 Discussion

In China, tinea capitis is not a notifiable infectious disease; therefore, it is not subject to mandatory reporting, and the isolation of dermatophytes from clinical samples does not require notification. In the absence of a systematic national surveillance system, the true disease burden and evolving epidemiology of tinea capitis are primarily revealed through targeted studies such as this one. Against this background, our study yielded the following key findings: (1) a marked increase in adult cases, especially among postmenopausal women; (2) a shift in predominant pathogens from anthropophilic to zoophilic dermatophytes; (3) distinct clinical types and risk factors between children and adults, indicating differences in host immunity and transmission patterns; (4) a high rate of household transmission with complete pathogen concordance.

Consistent with global epidemiological patterns, children remain the most affected group (71.13%), particularly those aged 6–12 years [10, 11]. The elevated susceptibility inherent to this population is multifactorial, attributable to immature immunity systems, underdeveloped skin barrier functions, insufficient production of antifungal sebum lipids, and high rates of close contact in group settings such as schools [12]. The substantial rise in adult cases (28.9% in the current study vs. 9.7% a decade ago, a trend corroborated elsewhere [13]), and the extreme female predominance in adults (F:M = 33.5:1) compared to the slight female preponderance in children (1.39:1), suggest different leading risk factors.

The slight female bias observed in children might be associated with longer hair, potentially facilitating pathogen harborage and posing challenges for thorough cleansing. In contrast, the overwhelming female predominance in adults, particularly among elderly postmenopausal women, strongly implicates the involvement of endocrine factors. A marked decline in postmenopausal oestrogen levels leads to reduced sebum production, consequently compromising the scalp's innate defences and facilitating fungal colonisation [14, 15]. Additionally, adult women are often the primary caregivers, potentially resulting in heightened exposure to infected children or domestic animals, representing a significant behavioural risk factor. This rise in adult cases, especially among women, is consistent with reports from Korea and other regions of China [16, 17], but contrasts with the epidemiology in many Western countries, where *M. canis* or *T. tonsurans* predominate and children constitute the vast majority of cases [18, 19, 20].

Pathogen spectra differed significantly between adults and children. Among children, although anthropophilic *T. violaceum* remained common (34.67%), zoophilic species (e.g., *M. canis* , 32.00%; *T. mentagrophytes*, 13.33%) collectively accounted for 45.33% of cases, indicating mixed transmission sources. The active behaviour of children and their frequent close contact with pets elevate the risk of acquiring zoophilic infections. Conversely, adults were predominantly infected with anthropophilic species (96.88%), mainly *T. violaceum* (67.19%), suggesting person‐to‐person transmission, often through caregiving or fomite contact [21, 22].

The trend towards zoophilic agents (e.g., *M. canis* prevalence increased from 2.4% a decade ago to 22.4% in the current study), which aligns with epidemiological transitions reported in some African and Asian countries [23, 24, 25], indicates a transition in Nanchang from an anthropophilic‐endemic region to a mixed anthropophilic‐zoophilic endemic area. This trend is likely driven by increasing urbanisation and the increasing prevalence of pet ownership [26, 27], although the local pathogen spectrum still differs from that of most other regions in China, where *M. canis* exhibits absolute dominance [8, 27, 28, 29, 30].

The high prevalence of kerion (42.94%) and grey patch (23.53%) in children is associated with their higher rate of zoophilic infections, which provoke intense inflammatory responses, and their heightened inflammatory tendency [31]. The predominance of black dot in adults (81.16%) corresponds to their near‐exclusive infection with anthropophilic dermatophytes, which are typically associated with milder, noninflammatory endothrix infections [31]. Risk factor analysis further underscores these behavioural differences: animal contact was a significant risk factor among children (55.29% vs. 34.78% in adults, *p* = 0.004), confirming the importance of exposure source.

The high household transmission rate (22.90%), combined with the identification of asymptomatic carriers (exclusively adults) in 50.00% of the affected households, suggests that adult cases are likely underreported and that adults serve as critical reservoirs and vectors for sustaining and propagating chains of infection [32, 33]. The observed 100% pathogen concordance within households provides strong evidence supporting intra‐household transmission.

Based on our findings, we recommend implementing a multifaceted and integrated public health strategy. For children, where transmission occurs via both anthroponotic and zoonotic routes, targeted health education initiatives within schools and communities are essential. Key messages should emphasise avoiding the sharing of personal items such as combs, hats, towels, and pillows. Promoting responsible pet ownership practices is crucial, including scheduling regular veterinary check‐ups and initiating prompt antifungal treatment if dermatophytosis is detected. Children must be educated on practicing thorough hand hygiene following animal contact and on avoiding close contact with animals showing skin lesions. Furthermore, enhancing hygiene management in educational settings (e.g., kindergartens and schools) is recommended, which may include implementing screening measures and environmental disinfection in classrooms where cases are identified.

For adults, especially elderly women, control strategies should focus on interrupting human‐to‐human transmission. Since clinical presentations in adults are often mild or atypical, frequently leading to misdiagnosis [34, 35], there is an urgent need to enhance clinician awareness and recognition of tinea capitis in this population to reduce both misdiagnosis and underdiagnosis. Furthermore, adult caregivers in households with infected children or pets should be informed of their potential risk, advised to perform regular self‐examinations, and instructed to seek prompt medical attention if symptoms such as hair loss or scaling appear.

Another crucial but often overlooked measure is screening all household close contacts of every confirmed index case, utilising direct microscopy and/or fungal culture. All symptomatic cases identified within a household should receive concurrent treatment until mycological cure is achieved. For asymptomatic carriers, the use of topical antifungals or medicated shampoos containing ketoconazole or selenium sulfide 2–3 times per week is recommended to eradicate the pathogen and prevent transmission [33]. Fomites such as combs, hats, pillows, and bedding used by patients should be disinfected by boiling, chemical soaking, or exposure to sunlight. Furthermore, barbering tools must also be thoroughly sterilised.

This study has several limitations. First, the single‐center, retrospective study conducted at a provincial tertiary hospital may introduce selection bias, which may have led to an overestimation of the proportion of severe cases and an underestimation of milder cases or those from socioeconomically disadvantaged or remote rural areas. Second, information on risk factors (e.g., contact with animals or infected individuals) was based on recall and is therefore subject to recall bias. Third, the household transmission analysis was performed on a subset of cases rather than a random sample, potentially introducing selection bias and resulting in an underestimation of the true household transmission rate and asymptomatic carriage prevalence. Fourth, and most crucially, the lack of molecular strain typing of isolates obtained from patients, their household members, and contacted animals precludes definitive confirmation of transmission chains and directions.

Future research should employ multi‐center, prospective study designs integrated with molecular typing techniques to simultaneously isolate and genetically compare pathogens obtained from patients, their close contacts, and contacted animals. This approach will enable the precise mapping of transmission networks, clarify the exact infection sources for different populations, and provide higher‐level evidence for formulating targeted interventions.

In conclusion, this study reveals significant differences between children and adults with tinea capitis in Nanchang in terms of gender distribution, pathogen spectrum, clinical manifestations, and risk factors. Our findings highlight emerging trends, including a rise in adult cases (particularly among elderly women) and a shift towards zoophilic pathogens. These findings necessitate precise, population‐specific interventions that target the particular sources and transmission routes relevant to each demographic. Future control efforts should integrate human and veterinary medicine by adopting a comprehensive control strategy—encompassing health education, active screening, environmental management, pet healthcare, and targeted treatment—to effectively curb the transmission and prevalence of tinea capitis.

## Author Contributions

**Qian Peng:** writing – original draft, formal analysis. **Afang Xu:** writing – review and editing. **Qijing Xiao:** writing – review and editing. **Zhihua Li:** methodology. **Qing Jiang:** methodology. **Yangmin Gao:** formal analysis. **Yun Jin:** formal analysis. **Yunpeng Luo:** investigation, data curation. **Xinyi Fan:** data curation, investigation. **Rui Xu:** investigation, data curation. **Jiao Xu:** investigation, data curation. **Wenjin Ai:** investigation, data curation. **Xiaobing Wang:** conceptualization, methodology.

## Conflicts of Interest

The authors declare no conflicts of interest.

## Supporting information


**Table S1:** myc70142‐sup‐0001‐Supinfo.docx.

## Data Availability

The data that support the findings of this study are available from the corresponding author upon reasonable request.
