# Supplementary material for: Epidemiology and Pathogen Shift of Tinea Capitis: A Comparative Analysis of Adults and Children in Nanchang, China (2022–2024)
Source: Mycoses. 2025 Dec 22;68(12):e70142. doi: 10.1111/myc.70142 (PMC12720223; doi:10.1111/myc.70142)
Supplement: Supplementary file 1 — Table S1: myc70142‐sup‐0001‐Supinfo.docx. [file MYC-68-e70142-s001.docx]

Table S1. Comparison of clinical types caused by the same pathogen between children and adults

| Pathogen | Clinical type | Children (n=150), n (%) | Adults (n=64), n (%) | P-value |
| --- | --- | --- | --- | --- |
| *T.violaceum* | Grey pacth | 2 (1.33) | 0 (0.00) | 0.475 |
|  | Black dot | 39 (26.00) | 36 (56.25) |  |
|  | Kerion | 11 (7.33) | 7 (10.94) |  |
| *T.tonsurans* | Grey pacth | 2 (1.33) | 0 (0.00) | 0.230 |
|  | Black dot | 12 (8.00) | 10 (15.63) |  |
|  | Kerion | 6 (4.00) | 1 (1.56) |  |
| *T.rubrum* | Grey pacth | 0 (0.00) | 0 (0.00) | 0.138 |
|  | Black dot | 1 (0.67) | 5 (7.81) |  |
|  | Kerion | 5 (3.33) | 3 (4.69) |  |
| *M.canis* | Grey pacth | 33 (22.00) | 0 (0.00) | N/A |
|  | Black dot | 0 (0.00) | 0 (0.00) |  |
|  | Kerion | 15 (10.00) | 0 (0.00) |  |
| *T.mentagrophytes* | Grey pacth | 1 (0.67) | 0 (0.00) | 0.177 |
|  | Black dot | 0 (0.00) | 1 (1.56) |  |
|  | Kerion | 19 (12.67) | 1 (1.56) |  |
| *N.gypsea* | Grey pacth | 1 (0.67) | 0 (0.00) | N/A |
|  | Black dot | 0 (0.00) | 0 (0.00) |  |
|  | Kerion | 3 (2.00) | 0 (0.00) |  |

† N/A: Not applicable due to zero cells in the adult group.
